# Assessment of the anti-virulence potential of extracts from four plants used in traditional Chinese medicine against multidrug-resistant pathogens

**DOI:** 10.1186/s12906-020-03114-z

**Published:** 2020-10-19

**Authors:** Zhonghui Pu, Huaqiao Tang, Nana Long, Min Qiu, Mingxiang Gao, Fenghui Sun, Min Dai

**Affiliations:** 1 School of Laboratory Medicine, Chengdu Medical College, Chengdu, Sichuan People’s Republic of China; 2 Laboratory of Veterinary Drug Residue Prevention and Control Technology of Animal-derived Food, Chengdu Medical College, Chengdu, Sichuan People’s Republic of China; 3 School of Clinical Medical Sciences, Chengdu Medical College, Chengdu, Sichuan People’s Republic of China

**Keywords:** TCM plants, Quorum sensing inhibition, Virulence, Biofilm

## Abstract

**Background:**

Multidrug-resistant pathogens are resistant to many antibiotics and associated with serious infections. *Amomum tsaoko* Crevost et Lemaire*, Sanguisorba officinalis, Terminalia chebula* Retz and *Salvia miltiorrhiza Bge*, are all used in Traditional Chinese Medicine (TCM) against multidrug-resistant pathogens, and the purpose of this study was to evaluate the antibacterial and anti-virulence activity of extracts derived from them.

**Methods:**

The antibacterial activity of ethanol and aqueous extracts from these four plants was examined against several multi-drug resistant bacterial strains, and their anti-virulence potential (including quorum quenching activity, biofilm inhibition, and blocking production of virulence factor δ-toxin) was assessed against different *S. aureus* strains. The chemical composition of the most effective extract was determined by LC-FTMS.

**Results:**

Only extracts from *S. officinalis* and *A. tsaoko* were shown to exhibit limited growth inhibition activity at a dose of 256 μg·mL-1. The *S. officinalis* ethanol extract, the ethanol and aqueous extract of *A. tsaoko*, and the aqueous extract of *S. miltiorrhiza* all demonstrated quorum quenching activity, but didn’t significantly inhibit bacterial growth. The ethanol extract of *S. officinalis* inhibited bacterial toxin production and biofilm formation at low concentrations. Chemical composition analysis of the most effective extract of *S. officinalis* showed that it mainly contained saponins.

**Conclusions:**

The most active extract tested in this study was the ethanol root extract of *S. officinalis*. It inhibited δ-toxin production and biofilm formation at low concentrations and saponins may be its key active components. While the four plants showed no direct antibacterial effects, their anti-virulence properties may be key to fighting bacterial infections.

**Supplementary information:**

The online version contains supplementary material available at 10.1186/s12906-020-03114-z.

## Background

The advent of antibiotics in the early twentieth century greatly reduced the mortality associated with infection. However, overuse or abuse of antibiotics has led to bacterial resistance becoming a serious global public health problem [1]. Drug-resistant bacterial infections have a huge burden on health care systems, veterinary practices, and society in general, and have an impact on a wide range of sectors, from farms to public health [2]. The antibiotic resistance levels of a large number of drug-resistant pathogens, including *Enterobacteriaceae*, *Campylobacter*, and *Candida*, are increasing [3]. Some bacteria are, or are about to be become, resistant to almost all antibiotics, such as the Carbapenem-resistant *Enterobacteriaceae* and the multi-drug resistant *Acinetobacter* [4]. The consequences of drug-resistant bacteria infections are serious and can include an extended duration of illness, longer hospital stays, higher mortality rates, and increased hospitalization costs [5]. Bacterial resistance to antibiotics not only threatens health, but also brings economic losses.

Antibiotic abuse, including incorrect usage, inaccurate dosages, frequency of use, and improper treatment, will lead to bacterial resistance and eventually promote the emergence of super bacteria [6]. Fluoroquinolones are used in large quantities in China, and resistance to these antibiotics has reached 60% [7]. In the past, skin and visceral infections caused by *S.aureus* were effectively treated with penicillin, but methicillin-resistant *Staphylococcus aureus* has now become the dangerous pathogens in nosocomial infections [8]. After carbapenem was introduced to China it became one of the main antibacterial drugs used to treat severe bacterial infections, but recently, there is an increasing in the number of carbapenem-resistant bacteria, with the most common being *Escherichia coli, Staphylococcus aureus*, *Klebsiella pneumoniae* and *Streptococcus pneumoniae*, followed by *Salmonella* [9]. A combination of the decline in the antibacterial efficacy of antibiotics and a lack of research on new antibiotics, means that a new anti-infection strategy should be established, and the anti-virulence method is thought to be a potentially effective way [10].

Traditional Chinese medicine (TCM) is a unique natural resource in China, with a long history in preventing and treating infectious diseases [11, 12]. The biggest feature and advantage of its anti-infection medicine is that it exerts anti-infective effects at an overall level. While TCMs have anti-infective properties, many also have antipyretic and anti-inflammatory effects [13]. TCMs anti-infective properties are strengthened by their functional enhancement of the body’s immune system [14]. Studies have shown that the bacteriostatic effect of TCM is related to the molecular structure of its active ingredients [15]. However, there have been minimal studies on the use of TCM to combat infection through anti-virulence mechanisms [16].

In this study, we selected four plants commonly used in TCM; *Amomum tsaoko* Crevost et Lemaire*, Sanguisorba officinalis, Terminalia chebula* Retz, and *Salvia miltiorrhiza Bge*. We examined the antibacterial activity of ethanol and aqueous extracts from these plants against the following multi-drug resistant bacterial strains, *A. baumannii, E. aerogenes, E. cloacae, E. faecium, K. pneumoniae, P. aeruginosa,* and *S. aureus*, and tested their anti-virulence potential against various *S. aureus* strains. We used *S. aureus* biosensor strains to assess the quorum sensing inhibition potential of the extracts, and to evaluate the δ-toxin synthesisas and biofilm formation inhibition effects of the most active extracts*.*

## Methods

### Preparation of the different extracts

Four plants commonly used in TCM (fruits of *Amomum tsaoko* Crevost et (#:20180705)*,* root of *Sanguisorba officinalis* (#:1811096)*,* fruits of *Terminalia chebula* (#:1811035) and root of *Salvia miltiorrhiza* (#:1811035) were purchased from Sichuan Neo-Green Pharmaceutical Technology Development Co., Ltd. (Pengzhou, China), in September 2018 and identified by Prof. Min Li.

Material from *A.tsaoko, S. officinalis, T. chebula,* and *S. miltiorrhiza* were air-dried and ground into a powder. To prepare the ethanol raw extracts, the powder was macerated at a ratio of 1:10 (w / v) in 1 L flasks for 72 h in 70% ethanol with regular agitation. The macerate was filtered and stored, and the residual plant material was reprocessed in 70% ethanol. The two macerates were combined and evaporated under reduced pressure using a rotary evaporator at ≤40 °C to obtain a dark brown residue, which were dissolved in dH_2_O and shell frozen in a dry ice-acetone bath before being lyophilized. Dried extracts were stored in scintillation vials at − 20 °C. Each plant sample (30 g) was subjected to distilled water (1:10 ratio w/v) for 20 min on a hot plate, followed by centrifugation and vacuum filtration to obtain aqueous extracts. The all extracts were dissolved to 10 mg mL^−1^in DMSO for biological assays.

### Bacterial strains and culture conditions

The multi-drug resistant bacteria used in this study (Table 2 and Table S1); *Acinetobacter baumannii* (EU-24, CDC-33), *Enterobacter aerogenes* (CDC-7) and *Enterobacter cloacae* (CDC-32); *Enterococcus faecium* (EU-49, EU-44), *Klebsiella pneumoniae* (EU-32, CDC-76), *Pseudomonas aeruginosa* (PAO1, CDC-54), and *Staphylococcus aureus* (AH845, NRS249, NRS232, NRS252), which were stored in Quave’s Lab (Emory University, Full details in Supplementary Table S1). All strains were grown on trypticase soy agar (TSA) plates and incubated at 37 °C for 12 h, then single colonies were picked and cultured in either Cation-adjusted Mueller Hinton II broth (CAMHB) or tryptic soy broth (TSB). All antibacterial tests were repeated three times and multiple independent experiments performed.

### Growth inhibition analyze

Extracts were screened for growth inhibition activity as previously reported [17]. Overnight liquid cultures grown in CAMHB were diluted to a confluence of 5 × 10^5^ CFU mL^− 1^ as determined by on their optical density (OD_600_). Assays were conducted in 96-well plates (CELLSTAR 655–185). Plates were incubated for 24 h and the OD_600_ were read by a Cytation-3 multimode plate reader (Biotek), after which, the percentage of growth inhibition was calculated. The OD was also used to calculate the IC_50_, and where the IC_50_ ≤ 256 μg·mL^− 1^, the dose-dependent anti-bacterial activity of the extract was tested.

### Agr reporters were used for quorum sensing assay

The quorum sensing inhibition activity of the extracts were evaluated by using the accessory gene regulator (agr) reporters of *S. aureus*, including AH1672, AH430, AH1747, and AH1872, according to previously described [10]. Quorum sensing inhibition activity of the extracts was equal to the reporter strains fluorescent protein (YFP) signal. The agr reporter strains were grown in TSA and maintained TSB, supplemented with chloramphenicol (10 μg·mL^− 1^). All anti-quorum sensing assays were conducted in black 96-well microtiter plates (Costar 3603, final well volume: 200 μL). Plates were incubated in a humidified shaker at 37 °C at 1200 rpm (Stuart SI505). The OD_600_ and fluorescence (493 nm excitation, 535 nm emission) were measured by a plate reader at 0 h and 22 h incubation (BioTek Cytation3). The initial concentration of 256 μg mL^− 1^ were selected to against *agr* 1–4 reporter strains. Dose response curves were tested by 2-fold serial dilutions method for a final concentration range from 2 to 256 μg mL^− 1^.

### Quantification of δ-toxin production

The most active extracts determined by the *agr* reporter assay were selected to against the δ-toxin production of *S. aureus* strains (AH1262 and NRS249) as previously described [18]. The sub-inhibitory concentrations of the extracts with a final volume of 1.5 mL were used in the assay. The tubes were incubated at 37 °C/275 rpm for 15 h, then samples were collected after the OD_600_ determined. All the samples were centrifuged at 13,000 g for 5 min at 4 °C and supernatants were moved to a vial for δ –toxin quantification by HPLC. Formylated and deformylated δ-toxin were normalized for growth (OD_600_) during supernatant harvest [19].

### Biofilm inhibition assay

The anti-virulence potential of the most active extracts determined by the *agr* reporter assay were determined by their biofilm inhibiting activity, assessed using a crystal violet assay, as previously described [20]. Briefly, after culturing the bacterial biofilm in a microplate, the culture solution was removed, the plate washed with PBS and dried, and the 1% crystal violet solution was added. After incubating for 30 min, the excess dye was washed off with water, and the dye attached to the biofilm is dissolved with ethanol or acetic acid. The OD was measured at 590 nm with a microplate reader. *S. aureus* strains UAMS-1 and UAMS-929 (isogenic biofilm deficient mutant of UAMS-1) were used in the assay [21].

### Chemical characterization of extracts

*Officinalis* EtOH extracts*,* which was determined to be the most effective extract in terms of antibacterial, anti-virulence, and anti-biofilm activities*,* was characterized by LC-FTMS using a Thermo Scientific LTQ-FT Ultra mass spectrometer equipped with a Shimadzu SIL-ACHT auto sampler and Dionex 3600SD HPLC pump. An Agilent Eclipse XDB column (250 × 4.6 mm) packed with C18 (5 μm) was used for analysis. Extracts were dissolved to10 mg mL^− 1^ in MeOH and passed through 0.45 μm filters and then 20 μL sample was injected by autosampler. Gradient elution was carried out with the solvent system A (0.1% formic acid) and system B (0.1% formic acid in ACN) at a flow rate of 1 mL min^− 1^, and the gradient steps were set from 98% A to 90% A in 19 min, to 85% A in 40 min, to 100% A in 66 min, before returning the column to initial conditions 98% A in 9 min. For all samples, data was collected from 190 to 600 nm using a diode array detector. MS data were acquired in MS1 mode in negative ESI (electrospray ionization) and positive ESI (electrospray ionization) mode at 150–1500 m / z and processed using Thermo Scientific Xcalibur 2.2 SP1.48 (San Jose, California). The capillary temperature of the negative and positive modes was 275.0 °C, 60 sheath gas, source voltage of 5.00 kV, source current of 100.0 μA, and capillary voltage of − 19.0 or + 32.0 V. The presumed formula of extract components was determined using X-caliber software to perform isotope abundance analysis of the high-resolution mass spectrometry data and report the best matching empirical formula. Database searches were performed using Scifinder (American Chemical Society) and Natural Product Dictionary (Taylor & Francis Group) and compounds with molecular masses corresponding to the above LC-FTMS data which had been previously identified from the same plant species were reviewed.

### Statistical analysis

All of the data were analyzed with a two-tailed Student’s t-test by GraphPad Prism 8.0 (GraphPad Software, La Jolla, CA). Cultures treated with DMSO or dH2O were used as vehicle controls, which were compared to extracts treated samples for all statistical analyses. *p < 0.05 or ** *p* < 0.01 was considered to be statistically significant.

## Results

### Limited growth inhibitory activity was confirmed in ESKAPE pathogens

To determine their growth inhibition activity, crude extracts (Table 1) were screened against each ESKAPE pathogen at 256 μg·mL^− 1^. Dose–response experiments were performed for extracts when the inhibition percentage above 40 for any individual strain. After the initial library screen, the EtOH and aqueous *S. officinalis* extracts and the EtOH *A. tsaoko* extracts showed significant activity and were therefore evaluated from further assay. The inhibitory activity of the EtOH and aqueous extracts is shown in Table 2.

**Table 1:** The parts used, extract ID, extraction solvent and yields of the plant species

Plant species	Part used	Extract ID	Extract solvent	Yield (%)
*T. chebula*				
	Fruits	CDY 1	EtOH	7.64
	Fruits	CDY 2	dH_2_O	9.35
*S. officinalis*				
	Root	CDY 3	EtOH	10.46
	Root	CDY 4	dH_2_O	7.82
*A. tsaoko*				
	Fruits	CDY 5	dH_2_O	7.56
	Fruits	CDY 6	EtOH	9.38
	Fruits peel	CDY 7	dH_2_O	8.93
*S. miltiorrhiza*				
	Root	CDY 8	dH_2_O	8.25
	Root	CDY 9	EtOH	7.84

**Table 2:** The growth inhibition results of selected ESKAPE pathogens by four medicinal plant samples

Plant Species	Extract ID	*A. baumannii*		*E. aerogenes*	*E. cloacae*	*E. faecium*		*K. pneumoniae*		*P. aeruginosa*		*S. aureus*			
		CDC-33	EU-24	CDC-7	CDC-32	EU-44	EU-49	EU-32	CDC-76	CDC-54	PAO1	AH845	NRS249	NRS232	NRS252
*T. chebula*	CDY 1	–	–	–	–	–	–	–	–	–	–	–	–	–	–
	CDY 2	–	–	–	–	–	–	–	–	–	–	–	–	–	–
*S. officinalis*	CDY 3	+	–	–	–	–	–	–	–	–	–	+	–	–	–
	CDY 4	+	–	–	–	–	–	–	–	–	–	–	–	–	–
*A. tsaoko*	CDY 5	–	–	–	–	–	–	–	–	–	–	–	–	–	–
	CDY 6	–	+	–	–	–	–	–	–	+	–	–	–	–	–
	CDY 7	–	–	–	–	–	–	–	–	–	–	–	–	–	–
*S. miltiorrhiza*	CDY 8	–	–	–	–	–	–	–	–	–	–	–	–	–	–
	CDY 9	–	–	–	–	–	–	–	–	–	–	–	–	–	–

Note: “+”: growth inhibition ≥40% vs. vehicle control; “−”: < 40% growth inhibition vs.vehicle control

### Active extracts exhibited dose related antibacterial activity

**Fig. 1:**
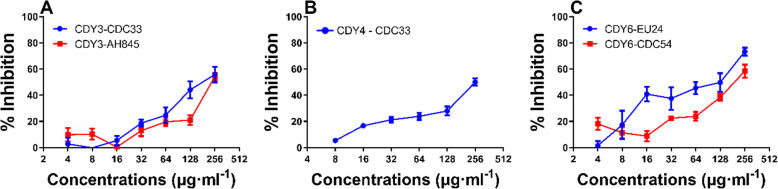
Extract mediated growth inhibition of selected ESKAPE pathogens. **a** Inhibition of CDC33 and AH845 by CDY3, **b** Inhibition of CDC33 by CDY4, **c** Inhibition of EU24 and CDC54 by CDY6

As shown in Fig. 1, the *S. officinalis* ethanol extract inhibited *Acinetobacter baumannii strain* (CDC-33) and *S. aureus* (AH845) growth with an IC_50_ of 256 μg·mL^− 1^. The *S. officinalis* aqueous extract inhibited *A. baumannii strain* (CDC-33) with an IC_50_ of 256 μg·mL^− 1^ and the ethanol extract inhibited *A. baumannii strain* (EU-24) and *P. aeruginosa strain* (CDC-54), with an IC_50_ of 128 and 256 μg·mL^− 1^ respectively.

### Extracts exhibited quorum quenching activity in *S. aureus*

**Fig. 2:**
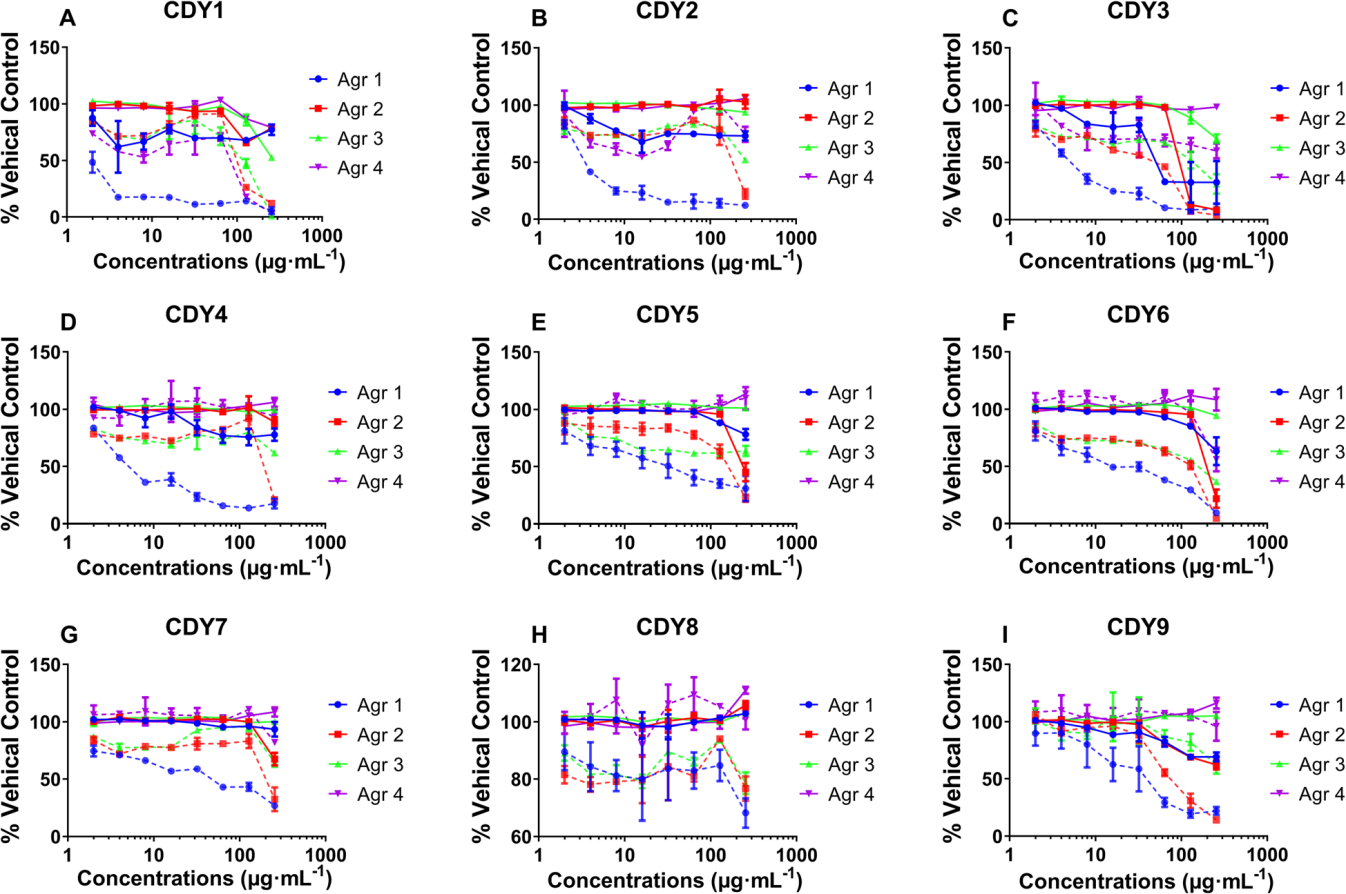
Quorum quenching activity of extracts. **a-i** quorum quenching activity of CDY1–9 on four *S. aureus* strains. Data are represented as percent of anti-agr activity or growth of the vehicle (DMSO) control at 24 h against the following strains: AH1677 (agr 1), AH430 (agr 2), AH1747 (agr 3), and AH1872 (agr 4). The dashed lines represent anti-agr activity (fluorescence), and the solid lines represent growth inhibition activity (OD_600_)

In *S. aureus*, the accessory gene regulator (agr) system plays an important role in the production of virulence factors by quorum-sensing component pathways [22]. The four allelic groups on the agr gene locus, agr I–IV [23], have been confirmed by genetic and agr-inhibiting methods [24]. We screened the quorum quenching activity of these extracts against *S. aureus* and the results are shown in Fig. 2. The *S. officinalis* EtOH extract exhibited dose dependent quorum quenching activity against Agr 1–4, but showed no anti-bacterial activity against Agr 2–4. The organic and aqueous extracts of *A. tsaoko* showed no anti-bacterial activity against the Agr strains, but significantly inhibited the quorum sensing of Agr 1–3. All other extracts, except for the *S. miltiorrhi* aqueous extract*,* showed quorum quenching activity against Agr 1.

### Extracts block production of virulence factor δ-toxin in *S. aureus*

**Fig. 3:**
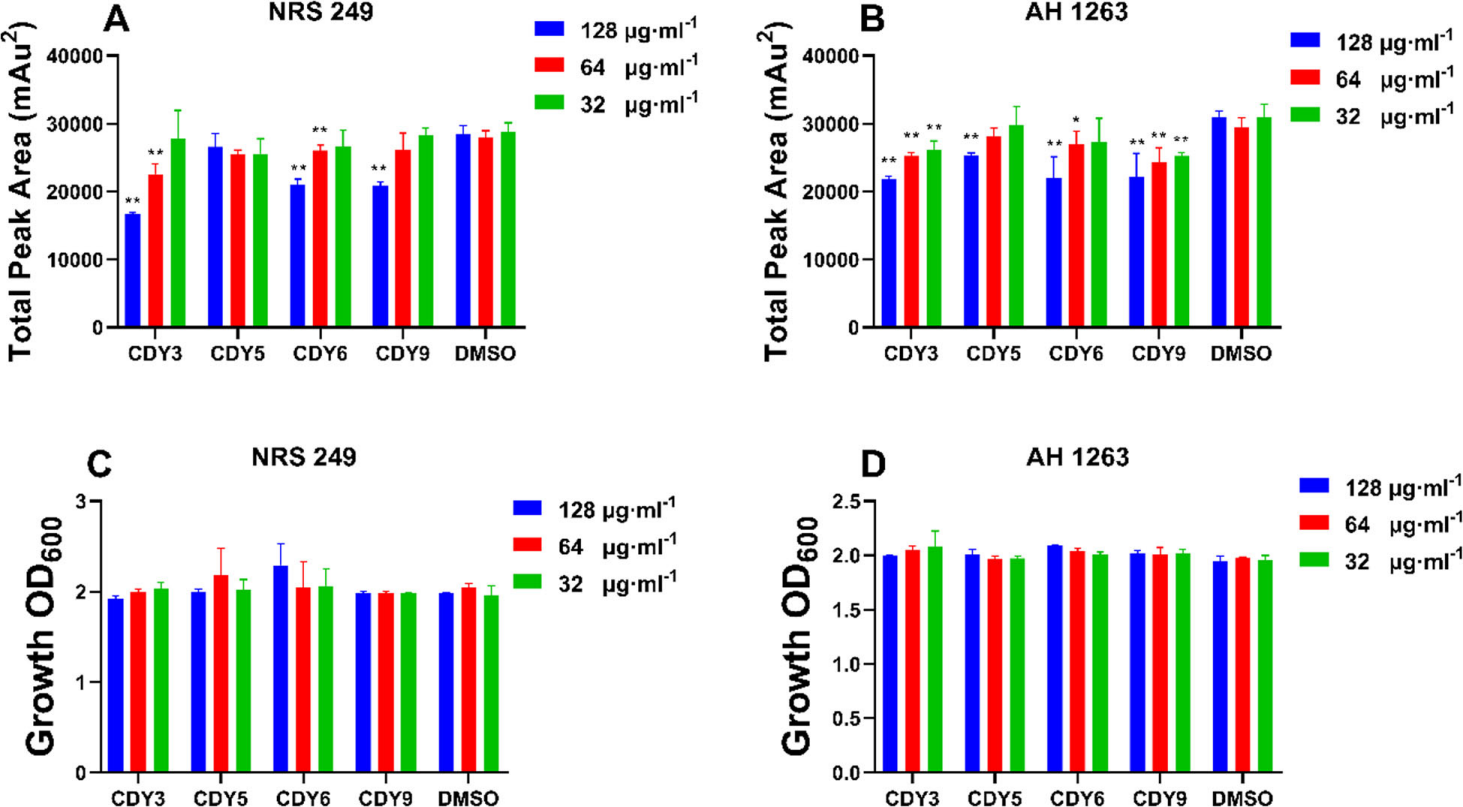
Extracts block δ-toxin production without exhibiting antibacterial activity. **a, b** CDY3, 5, 6 and 9 inhibition of δ-toxin production in NRS249 and AH1263, **c, d** CDY3, 5, 6 and 9 inhibition of NRS249 and AH1263 growth. * *p* < 0.05 or ** *p* < 0.01 vs. DMSO

The strains AH 1263 and NRS 249 produce high levels of δ-toxin. Toxin levels were measured after strains were treated with CDY 3, 5, 6, and 9 (extracts which exhibited quorum quenching activity). CDY3, 6 and 9 significantly inhibited δ-toxin production in *S. aureus* (NRS 249) and CDY 3, 5, 6, and 9 showed dose dependent inhibition of δ-toxin produced by *S. aureus*. All inhibitory effects were produced without inhibiting bacterial growth (Fig. 3). CDY 3 exhibited the most effective anti-δ-toxin activity at a concentration of 128 μg·mL^− 1^.

### Extracts exhibit inhibition of *S. aureus* biofilm

**Fig. 4:**
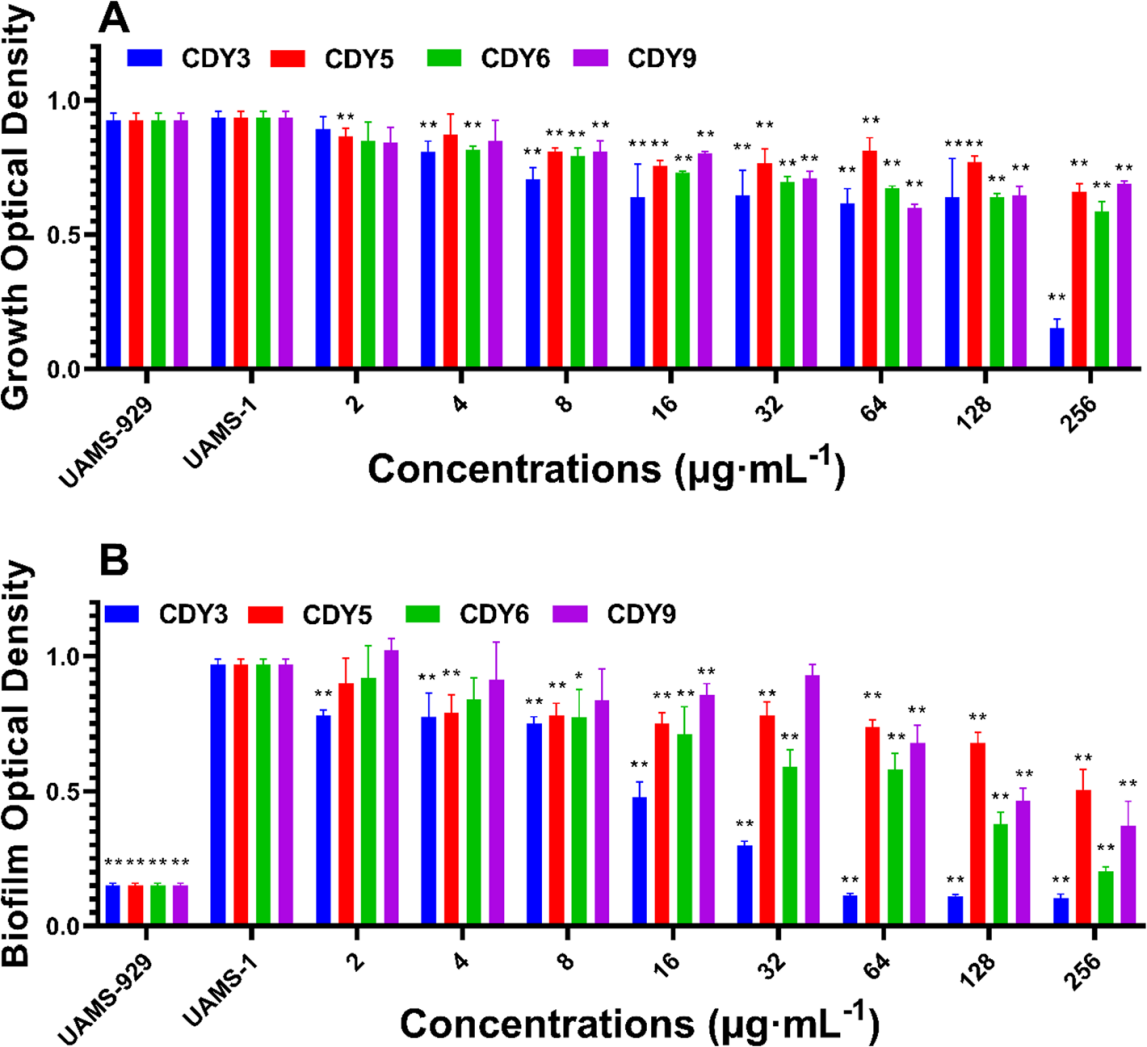
Inhibition of *S. aureus* biofilm formation by extracts. Growth and biofilm inhibition of CDY3, 5, 6, and 9 against UAMS-1. The OD 595 nm is plotted along with OD 600 nm, measured by transfer of the well supernatants to a new 96-well plate.* *p* < 0.05 or ** *p* < 0.01 vs. UAMS-1

The inhibition activity of the extracts against biofilm formation was tested with *S. aureus* strains UAMS-929 and UAMS-1. Sar is a winged helix transcription factor that can regulate biofilm formation. UAMS-929 has the *sar* gene knocked out and cannot produce biofilms and thus is used as a control. CDY3 showed the best biofilm inhibition activity in a dose related manner, CDY6 and CDY9 also inhibited biofilm formation, but only at higher doses, and CDY5 exhibited limited biofilm inhibition. The results are shown in Fig. 4.

### Chemical characterization of active extracts

**Fig. 5:**
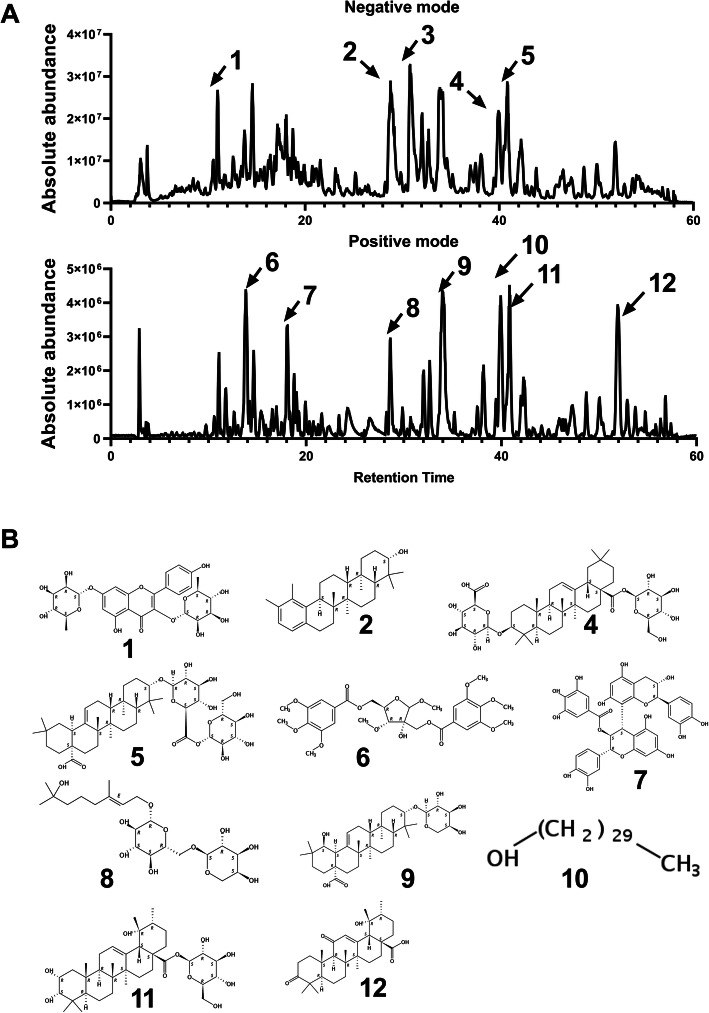
Chemical characterization of active components of CDY3

Characterization of the major constituents of the ethanol extract of *S. officinalis*. All peaks correspond to the data presented in Supplementary materials. Table S2 and S3, and putative structural matches are listed by peak number (Fig. 5). Peak 1 was determined to be C_36_H_58_O_9_ and putative structural matches include; Olean-12-en-28-oic acid, 3,19-dihydroxy-, β-D-glucopyranosyl ester, (3β,19α). Peak 2 was determined to be C_41_H_64_O_13_ and putative structural matches include; β-D-Glucopyranose, 1 - [(2S,4aS,4bR,6aR,8S,10aS,10bR) - 8 - (α-L-arabinopyranosyloxy) - 2,3,4,4a,4b,5,6,6a,7,8,9,10,10a,10b-tetradecahydro-4a,4b,7,7,10a-pentamethyl-2-[(3R)-3-methyl-4-oxopentyl]-2-chrysenecarboxylate]. Peak 5 was assayed to be C_36_H_56_O_12_ and putative structural matches include; Urs-12-ene-23,28-dioic acid, 2,3,19-trihydroxy-, 28-β-D-glucopyranosyl ester, (2α,3β,4α)-. Peak 6 was determined to be C_30_H_27_O_12_ and putative structural matches include; D-Ribofuranoside, methyl 3-O-methyl-2-C-[[(3,4,5-trimethoxybenzoyl) oxy] methyl]-, 5-(3,4,5-trimethoxybenzoate). Peak 9 was determined to be C_35_H_57_O_8_ and putative structural matches include; Olean-12-en-28-oic acid, 3-(α-L-arabinopyranosyloxy)-19-hydroxy-, (3β,19α)-. Peak 10 was determined to be C_30_H_62_O and putative structural matches include; 1-Triacontanol. Peak 11 was determined to be C_36_H_58_O_10_ and putative structural matches include; Urs-12-en-28-oic acid, 2,3,19-trihydroxy-, *β*-D-glucopyranosyl ester, (2α,3α)-. Peak 12 was determined to be C_30_H_45_O_5_ and putative structural matches include; Urs-12-en-28-oic acid, 19-hydroxy-3,11-dioxo-. This shows that the active ingredients of the EtOH extract mainly include saponins, flavonoids, phenolic glycosides, and lignins.

## Discussion

Traditional Chinese Medicine and its compounds have comprehensive therapeutic effects including inhibiting virus replication, preventing virus-induced cytopathy, and regulating immune function, as well as analgesic and anti-inflammatory properties. It therefore has unique advantages and broad development prospects for the prevention and treatment of infectious diseases [25]. However, many studies have shown that the anti-infective effect of Chinese medicine may not depend on its anti-bacterial properties, and some traditional medicines used to treat infections do not exhibit any antibacterial effects. In this study, only the organic and aqueous extracts of *S. officinalis* and the ethanol extract of *A. tsaoko* exhibited moderate anti-bacterial activity. The *S. officinalis* ethanol extract had better anti-bacterial activity than the aqueous extract [26] and was effective against both *A. baumannii* and *S. aureus*, whereas the aqueous extract was only active against *A. baumannii*. In previous studies, the *S. officinalis* extract exhibited weak antibacterial activity, and the polyphenolic ingredients possessed antibacterial activity with an MIC were from 0.78 to 25 mg/mL when tested against different pathogens, including Gram-negative bacteria (*Escherichia coli and Salmonella typhimurium*) and Gram-positive bacteria (*Staphylococcus aureus, Listeria monocytogenes and Bacillus subtilis*) [27]. The *A. tsaoko* ethanol extract exhibited anti-bacterial activity against *A. baumannii* and *P. aeruginosa*, whereas the aqueous extract did not. Most previous studies have examined the antibacterial activity of the extract or volatile oil of *A. tsaoko*, and although some results implied that they had antibacterial effects, this may be due to the larger concentrations used in these studies [28, 29]. *S. officinalis* is a TCM, which be used to treat hemorrhoids, wounds, and ulcers in Eastern Asian countries [30]. Previous research involving extraction and pharmacological studies of this herb have been carried out to assess its anti-viral, anti-inflammation, anti-bacterial, anti-tumor, and immunomodulation properties [31–33]. *A. tsaoko* is widely used in TCM to treat stomach disorders, malaria, throat infections, diarrhea, dyspepsia, nausea, and abdominal pain, and some researchers have reported it to have broad anti-bacterial, anti-tumor, and anti-inflammatory activity [34–36]. In this study, we have made the first evaluation the activity of these extracts on multidrug-resistant bacteria, and shown that they had limited growth inhibition activity. Some previous studies support our conclusion by showing that most of these extracts only have antibacterial activity when used at mg level concentrations.

Because these extracts exhibited limited anti-bacterial activity, this raises the question of why *A. tsaoko, S. officinalis, T. chebula,* and *S.miltiorrhiza* are still used in TCM to treat infectious diseases [37–39]. Recent studies have shown that information exchange occurs between bacteria. Many bacteria can synthesize and release a signaling molecule called autoinducer (AI), the extracellular concentration of which increases as bacterial density reaches a critical concentration. AI can active the expression of related genes and regulate the biological behavior of bacteria, including the production of toxins, biofilms, antibiotics, spores, and fluorescence to adapt to changes in the environment. This phenomenon is called quorum sensing [40]. Since this induction phenomenon occurs only after the bacterial density reaches a certain threshold, some people also call this phenomenon cell density dependent control of gene expression [41]. Quorum sensing is closely related to the infectious capacity and pathogenicity of bacteria, and so quorum sensing inhibitors have attracted increasing attention from drug researchers [41]. Research on quorum-sensing inhibitors is considered to be a powerful direction for the study of new antibacterial drugs [42]. We therefore evaluated the quorum quenching activity of the nine extracts on Agr1–4 reporter strains. Most extracts exhibited dose related quorum quenching activity on at least one Agr strain. The *S. officinalis* ethanol extract, the *A. tsaoko* organic and aqueous extracts, and the *S.miltiorrhiza* ethanol extract all exhibited quorum quenching activity without significantly inhibiting bacterial growth. A recent study evaluated the anti-QS activity of the *A. tsaoko* extract using *Chromobacterium violaceum* a biosensor strain. They showed that it exhibited anti-QS activity in a dose-dependent manner at a concentration range of 0.5–4 mg/mL [28]. Our study is the first one to use agr reporter strains to evaluate the quorum quenching activity of these extracts.

Quorum sensing were used by bacteria to coordinate certain behaviors, including biofilm formation, virulence, and resistance to antibiotics, depending on their local population density [43, 44]. The extracts with quorum quenching activity (CDY3, 5, 6 and 8) were further evaluated for their ability to inhibit toxin production and biofilm formation. The ethanol extract of *S. officinalis*, the aqueous extract of *A. tsaoko*, and the ethanol extract of *S. miltiorrhiza* all inhibited δ-toxin production in both *S. aureus* strains NRS 249 and AH1263 without affecting bacterial growth. All four extracts with quorum quenching activity also inhibited the biofilm formation in UAMS-1, with the ethanol extract of *S. officinalis* and the aqueous extract of *A. tsaoko* exhibiting better inhibition activity. The anti-quorum sensing and anti-biofilm activities of *A.tsaoko* on food borne pathogens have been reported [28]. The ethanol extract of *S. officinalis* has been shown to inhibit the biofilm formation of MRSA [45]. Some of the polyphenolic compounds in *S. officinalis* have strong anti-bacterial activity [46]. Some polyphenolic compounds were found in our results and play an important role in inhibiting quorum sensing.

## Conclusion

In conclusion, our study has shown that the ethanol extract of *S. officinalis* has considerable anti-infection capacity against five bacterial strains, and *S. aureus* in particular. It can inhibit bacterial toxin production and biofilm formation at low concentrations and does not promote bacterial proliferation, but can kill bacteria when used at high concentrations. The polyphenolic compounds were highly associated with its antibacterial and quorum quenching activities.. However, the underlying mechanisms still need to be better elucidated in further studies. This is the first comprehensive study examining the anti-virulence activities of the *S. officinalis* extract via its quorum quenching mechanism, and our results show that it has potential as a novel, natural, anti-infection medicine.

## Supplementary information

Additional file 1: **Table S1.** ESKAPE pathogens tested and their corresponding antibiotic resistance profiles as reported by the source provider. **Table S2.** Negative ESI Mass spectrometry (m/z) analysis of extract CDY3; peaks with > 0.5% relative abundance is listed. **Table S3.** Positive ESI Mass spectrometry (m/z) analysis of extract CDY3; peaks with > 0.5% relative abundance is listed.

## Data Availability

The data used to support the findings of this study are available from the corresponding author upon request.

## Abbreviations

WHO: World Health Organization
MRSA: Methicillin-resistant *Staphylococcus aureus*
agr: Accessory gene regulator
HPLC: High Performance Liquid Chromatography
