# Supplementary material for: Assessment of the anti-virulence potential of extracts from four plants used in traditional Chinese medicine against multidrug-resistant pathogens
Source: BMC Complement Med Ther. 2020 Oct 19;20:318. doi: 10.1186/s12906-020-03114-z (PMC7574281; doi:10.1186/s12906-020-03114-z)
Supplement: Supplementary file 1 — Table S1. ESKAPE pathogens tested and their corresponding antibiotic resistance profiles as reported by the source provider. Table S2. Negative ESI Mass spectrometry (m/z) analysis of extract CDY3; peaks with > 0.5% relative abundance is listed. Table S3. Positive ESI Mass spectrometry (m/z) analysis of extract CDY3; peaks with > 0.5% relative abundance is listed. [file 12906_2020_3114_MOESM1_ESM.docx]

**Assessment of the anti-virulence potential of extracts from four plants used in Traditional Chinese Medicine against Multidrug-Resistant Pathogens**

**Supplementary materials**

**Table S1 ESKAPE pathogens tested and their corresponding antibiotic resistance profiles as reported by the source provider**

| Species | Strain ID | Alternate ID | Antibiotic resistance profile* | Other characteristics |
| --- | --- | --- | --- | --- |
| Acinetobacter baumannii | CDC-33 | AR-BANK #0033 | CAZ, CIP, CRO, CTX, DOR, FEP, GEN, IPM, LVX, MEM, SAM, SXT, TOB, TZP | reduced susceptibility, elevated carbapenem MICs |
|  | EU-24 | Naval-81; NR-17786 | PIP, SXT, TET, TZP |  |
| Enterobacter aerogenes | CDC-7 | AR-BANK #0007 | AMC, AMP, ATM, CAZ, CFZ, CIP, CRO, CTX, ETP, FEP, FOX, LVX, SAM, TET | reduced susceptibility, elevated carbapenem MICs |
| Enterobacter cloacae | CDC-32 | AR-BANK #0032 | AMC, AMP, ATM, CAZ, CFZ, CRO, CTX, ETP, FEP, FOX, IPM, MEM, SAM, SXT, TZP | reduced susceptibility, elevated carbapenem MICs |
| Enterococcus faecium | EU-44 | HM-959; Strain 513 | AMC, RA, SXT, TET, TZP |  |
|  | EU-49 | NR-31915; Strain E0164 | AMC, GEN, TET, SXT, TZP, VAN |  |
| Klebsiella pneumoniae | EU-32 | NR-15410 | AMC, PIP, SXT, TZP | Contains b-lactamase K. pneumoniae carbapenemase (blaKPC) gene |
|  | CDC-76 | AR-BANK #0076 | AMC, AMP, CAZ, CFZ, CTX, DOR, FEP, FOX, GEN, IPM, MEM, SAM, SXT, TOB, TZP | reduced susceptibility, elevated carbapenem MICs |
| Pseudomonas aeruginosa | CDC-54 | AR-BANK #0054 | CAZ, FEP, GEN, IPM, LVX, MEM, TOB, TZP |  |
|  | PAO1 | AH-0071 |  |  |
| Staphylococcus aureus | LAC | AH0845 | OXA, PIP | PFGE: USA300; CA-MRSA |
|  | UAMS-1 |  |  | Osteomyelitis clinical isolate; prototype biofilm isolate |
|  | UAMS-929 |  |  | Isogenic 1sarA mutant of UAMS-1, biofilm deficient control strain |
|  | AH1677 |  |  | agr I yfp reporter strain (chloramphenicol resistant) |
|  | AH430 |  |  | agr II yfp reporter strain |
|  | AH1747 |  |  | agr III yfp reporter strain |
|  | AH1872 |  |  | agr IV yfp reporter strain |
|  | NRS232 | HT20020065 | ERY , GEN, PEN | agr I, egc+, (lukS-lukF PVL)+, hlg+; associated with necrotising pneumonia |
|  | NRS242 | HT20020238 | ERYI , PEN | agr IV, egc+, (lukS-lukF PVL)+, (lukE-lukD)+, hlgv+; associated with impetigo |
|  | NRS249 | HT20020341; NR-46042 | CIP, CLI, ERY, GEN, OXA, PEN | agr I, sea+, (lukE-lukD)+, hlgv+ associated with native valve endocarditis; SCCmec type IV |
|  | NRS385 | 95938; NR-46071 | CIP, CLI, ERY, GEN, LVX, SXT, TET | agr I; PFT is USA500, MLST is ST8, spa type is YHGCMBQBLO, SCCmec IV; sea+, seb+; HA-MRSA |

* Resistance: AMC, amoxicillin-clavulanic acid; AMP, ampicillin; ATM, aztreonam, CAZ, ceftazidime; CFZ, cefazolin; CIP, ciprofloxacin; CLI, clindamycin; CRO, ceftriaxone; CTX, cefotaxime; DOR, doripenem; ETP, ertapenem; FEP, cefepime; FOX, cefoxitin; GEN, gentamicin; IPM, imipenem; LVX, levofloxacin; MEM, meropenem; OXA, oxacillin, PEN, penicillin; PIP, piperacillin; RA, rifampicin; SAM, ampicillin-sulbactam; SXT, trimethoprim-sulfamethoxazole; TET, tetracycline; TOB, tobramycin; TZP, piperacillin-tazobactam; VAN, vancomycin. Any antibiotics denoted with an I indicates intermediate resistance.

**Table S2 Negative ESI Mass spectrometry (m/z) analysis of extract CDY3; peaks with >0.5% relative abundance is listed.**

| **RT (min)** | **Absolute Abundance** | **Formula (Δ-ppm)** | **Putative Compounds (Scifinder)** | **CAS** | **m/z  ([M-H]- unless indicated)** | **MS^2^** |
| --- | --- | --- | --- | --- | --- | --- |
| 3.06 | 2.34 | C_17_H_24_O_10_ (0.866) | β-​D-​Glucopyranoside, 4-​[3-​(hydroxymethyl)​-​2-​oxiranyl]​-​2,​6-​dimethoxyphenyl | 623928-18-7 | **387.11528**; 549.16975 | 340.93381; 322.83691 |
| 3.78 | 0.9 | C_30_H_42_O_5_ (-1.998) | Ursa-​12,​17,​19,​21-​tetraen-​23-​oic acid, 2,​3,​27-​trihydroxy-​, methyl ester, (2α,​3β,​4α)​- | 1172584-63-2 | **481.06389**; 515.07040 | 300.94851; 419.28163 |
| 6.67 | 0.86 | C_13_H_16_O_10_ (0.280); C_16_H_12_O_8_ (0.362) | D-​Ribose, 2-​C-​(hydroxymethyl)​-​, 5-​(3,​4,​5-​trihydroxybenzoate);  β-​D-​Glucopyranose, 1-​(3,​4,​5-​trihydroxybenzoate) ; Spiro[isobenzofuran-​1(3H)​,​9'-​[9H]​xanthen]​-​3-​one, 3',​6'-​dihydroxy-  4H-​1-​Benzopyran-​4-​one, 2-​(3,​4-​dihydroxyphenyl)​-​3,​5,​7-​trihydroxy-​6-​methoxy- | 90275-97-1;  13405-60-2;  2321-07-5;  519-96-0 | **331.06735**; 301.05668 | 168.91152; 270.98081 |
| 8.45 | 1.1 | C_19_H_26_O_3_ (1.523) C_14_ H_6_ O_8_  C_15_ H_10_ O_7_ | Androst-​4-​ene-​3,​17-​dione, 4-​hydroxy- ;  Benzopyrano[5,​4,​3-​cde]​[1]​benzopyran-​5,​10-​dione, 2,​3,​7,​8-​tetrahydroxy- ;  4H-​1-​Benzopyran-​4-​one, 2-​(3,​4-​dihydroxyphenyl)​-​3,​5,​7-​trihydroxy- | 566-48-3;  476-66-4;  117-39-5 | **301.05701**; 603.12181 | 300.99181; 585.40157 |
| 10.55 | 0.8 | C_48_H_19_O (-0.959) | Not match |  | 611.14318; 305.06744 | 305.03128; 219.05775 |
| 11 | 3.88 | C_46_H_27_O_7_ (0.224) | Not match |  | 691.17645; 345.08406 | 345.02822; 400.93237 |
| 11.68 | 0.69 | C_27_H_30_O_15_ (2.756) | 4H-​1-​Benzopyran-​4-​one, 7-​[[6-​O-​(6-​deoxy-​α-​L-​mannopyranosyl)​-​β-​D-​glucopyranosyl]​oxy]​-​3,​5-​dihydroxy-​2-​(4-​hydroxyphenyl)​- | 103102-81-4 | **593.13282**; 453.06896 | 441.03412; 423.05815 |
| 12.59 | 1.19 | C_36_H_58_O_9_ (-0.407) C_27_H_22_O_18_ | Olean-​12-​en-​28-​oic acid, 3,​19-​dihydroxy-​, β-​D-​glucopyranosyl ester, (3β,​19α)​-  Urs-​12-​en-​28-​oic acid, 3,​19-​dihydroxy-​, β-​D-​glucopyranosyl ester, (3β)​-  β-​D-​Glucopyranose, cyclic 2,​3-​[(1S)​-​4,​4',​5,​5',​6,​6'-​hexahydroxy[1,​1'-​biphenyl]​-​2,​2'-​dicarboxylate] 1-​(3,​4,​5-​trihydroxybenzoate)  α-​D-​Glucopyranose, cyclic 2,​3-​[(1S)​-​4,​4',​5,​5',​6,​6'-​hexahydroxy[1,​1'-​biphenyl]​-​2,​2'-​dicarboxylate] 1-​(3,​4,​5-​trihydroxybenzoate)  β-​D-​Glucopyranose, cyclic 4,​6-​[(1S)​-​4,​4',​5,​5',​6,​6'-​hexahydroxy[1,​1'-​biphenyl]​-​2,​2'-​dicarboxylate] 1-​(3,​4,​5-​trihydroxybenzoate) | 155653-86-4;  83725-24-0;  87392-62-9;  84316-77-8;  517-46-4 | 633.07644; 783.07453 | 300.93900; 463.05133 |
| 13.38 | 0.8 | C_27_H_27_O_27_ (-0.018) | Not match |  | 483.07990; 783.07450 | 300.93900; 463.05134 |
| 13.78 | 2.29 | C_30_H_26_O_12_ (2.341) C_27_H_30_O_14_ | [4,​8'-​Bi-​2H-​1-​benzopyran]​-​3,​3',​5,​5',​7,​7'-​hexol, 2,​2'-​bis(3,​4-​dihydroxyphenyl)​-​3,​3',​4,​4'-​tetrahydro-​, (2R,​2'R,​3S,​3'S,​4S)​-  4H-​1-​Benzopyran-​4-​one, 3,​7-​bis[(6-​deoxy-​α-​L-​mannopyranosyl)​oxy]​-​5-​hydroxy-​2-​(4-​hydroxyphenyl)​- | 23567-23-9; 482-38-2 | **577.13749**; 439.05717 | 425.03865; 407.11640 |
| 14.59 | 3.38 | C_30_H_27_O_12_ (2.881) | Not match |  | **579.15368**; 869.23276 | 289.00821; 245.07614 |
| 15.46 | 0.77 | C_30_H_25_O_12_ (1.361) | Not match |  | 577.13651; 635.09115 | 425.03865; 407.11640 |
| 16.82 | 1.34 | C_27_H_23_O_18_ (1.503) | Not match |  | 635.09049; 577.13766 | 609.17291; 611.05411 |
| 17.16 | 4 | C_18_H_30_O_2_ (-2.553) | 6,​9,​12-​Octadecatrienoic acid, (6Z,​9Z,​12Z)​- | 506-26-3 | **277.00231**; 555.01262 | 196.95436; 110.88324 |
| 18.06 | 1.61 | C_37_H_30_O_16_ (2.502) | Benzoic acid, 3,​4,​5-​trihydroxy-​, (2R,​2'R,​3S,​3'S,​4S)​-​2,​2'-​bis(3,​4-​dihydroxyphenyl)​-​3,​3',​4,​4'-​tetrahydro-​3',​5,​5',​7,​7'-​pentahydroxy[4,​8'-​bi-​2H-​1-​benzopyran]​-​3-​yl ester | 86631-41-6 | **729.14861**; 561.14254 | 577.05201; 559.14044 |
| 18.75 | 1.92 | C_32_H_48_O_5_ (1.226) | Urs-​19-​en-​28-​oic acid, 3,​12,​13-​trihydroxy-​, γ-​lactone, acetate (7CI) | 107965-02-6 | 511.24084; 551.04071 | 465.11229; 333.13311 |
| 19.91 | 0.59 | C_34_H_28_O_22_ (1.195) | D-​Ribofuranose, 2-​C-​[[(3,​4,​5-​trihydroxybenzoyl)​oxy]​methyl]​-​, 1,​3,​5-​tris(3,​4,​5-​trihydroxybenzoate) ; β-​D-​Glucopyranose, 1,​2,​3,​6-​tetrakis(3,​4,​5-​trihydroxybenzoate) ; D-​Glucose, 2,​3,​4,​6-​tetrakis(3,​4,​5-​trihydroxybenzoate) | 90276-00-9;  79886-50-3;  40410-95-5 | **787.10114**; 729.15049 | 617.04920; 635.08198 |
| 20.71 | 0.57 | C_32_H_48_O_5_ (1.256) | Urs-​19-​en-​28-​oic acid, 3,​12,​13-​trihydroxy-​, γ-​lactone, acetate (7CI) | 107965-02-6 | **513.25652**; 477.14198 | 467.13327; 335.22325 |
| 21.1 | 0.89 | C_35_H_56_O_8_ (-1.717); C_36_H_60_O_7_ | Olean-​12-​en-​28-​oic acid, 3-​(α-​L-​arabinopyranosyloxy)​-​19-​hydroxy-​, (3β,​19α)​- ; Urs-​12-​en-​28-​oic acid, 3-​(α-​L-​arabinopyranosyloxy)​-​19-​hydroxy-​, (3β)​- ; β-​D-​Glucopyranoside, (3β,​12β)​-​12-​hydroxydammara-​20,​24-​dien-​3-​yl | 866216-15-1;  35286-59-0;  364779-14-6 | **603.00705**; 300.99953 | 300.99181; 585.40157 |
| 21.58 | 1.01 | C_30_H_46_O_4_ (1.978); C_32_H_52_O_2_ | Benzo[3,​4]​-​18-​norandrosta-​3,​5,​15-​triene-​3(2'H)​-​carboxylic acid, 3',​4',​5',​6'-​tetrahydro-​3'-​hydroxy-​15-​(hydroxymethyl)​-​3',​4',​9,​14,​17,​17-​hexamethyl-​, (3β,​3'α,​4β,​4'α,​8α,​9β,​10α,​13α,​14β)​; Urs-​12-​en-​28-​oic acid, 19-​hydroxy-​3-​oxo- ; Benzoic acid, 3-​[(5,​10-​dihydro-​2,​3,​7,​8-​tetrahydroxy-​5,​10-​dioxo[1]​benzopyrano[5,​4,​3-​cde]​[1]​benzopyran-​1-​yl)​oxy]​-​4,​5-​dihydroxy- | 128397-09-1;  82203-11-0;  13849-90-6 | **469.05261**; 939.11329 | 393.15837; 769.04909 |
| 23.14 | 1.27 | C_11_H_7_O_16_ (-1.407) | Not match |  | **394.97255**; 315.01530 | 225.06172; 316.94911 |
| 25.21 | 0.62 | C_29_H_14_O (-1.317) | 28-​Norursa-​17,​19,​21-​trien-​3-​ol, (3β)​- | 1301266-38-5 | **408.98829**; 329.03115 | 328.98545; 289.10450 |
| 26.49 | 0.85 | C_24_H_23_O_15_ (1.167) | Not match |  | 551.10541; **836.59027** | 633.06579; 343.01016 |
| 28.26 | 0.14 | C_21_H_17_O_12_ (1.111) | Not match |  | **461.07366**; 923.15669 | 328.98388; 330.05620 |
| 28.81 | 8.65 | C_29_H_44_O (-1.167) | 28-​Norursa-​17,​19,​21-​trien-​3-​ol, (3β)​- | 1301266-38-5 | **408.98844**; 329.03093 | 328.98545; 289.10450 |
| 29.83 | 0.52 | C_41_H_13_O (-1.628) | Not match |  | 521.09556; 827.44618 | 343.00672; 399.04196 |
| 30.82 | 7.55 | C_13_H_11_O_16_ (-0.997) | Not match |  | **423.00426**; 343.04660 | 343.00480; 288.71902 |
| 32.07 | 2.58 | C_30_H_63_O_17_ (-1.793) | Not match |  | **695.40528**; 979.43879 | 487.33445; 649.03470 |
| 32.7 | 2.15 | C_41_H_64_O_13_ (-2.443) | β-​D-​Glucopyranose, 1-​[(2S,​4aS,​4bR,​6aR,​8S,​10aS,​10bR)​-​8-​(α-​L-​arabinopyranosyloxy)​-​2,​3,​4,​4a,​4b,​5,​6,​6a,​7,​8,​9,​10,​10a,​10b-​tetradecahydro-​4a,​4b,​7,​7,​10a-​pentamethyl-​2-​[(3R)​-​3-​methyl-​4-​oxopentyl]​-​2-​chrysenecarboxylate] ; Ursa-​12,​19(29)​-​dien-​28-​oic acid, 3-​(α-​L-​arabinopyranosyloxy)​-​23-​hydroxy-​, β-​D-​glucopyranosyl ester, (3β,​4α)​- (9CI) | 1442692-05-8;  356785-73-4 | 695.40623; **763.39460** | 487.33445; 649.03471 |
| 34.01 | 8.54 | C_35_H_71_O_20_ (-2.784) | Not match |  | **811.45167;** 1195.18011 | 603.39012; 765.05259 |
| 36.97 | 1.29 | C_20_H_18_O_14_ (-0.680) | D-​Glucose, cyclic 4,​6-​[(1S)​-​4,​4',​5,​5',​6,​6'-​hexahydroxy[1,​1'-​biphenyl]​-​2,​2'-​dicarboxylate] (9CI) ; D-​Glucose, cyclic 2,​3-​[(1S)​-​4,​4',​5,​5',​6,​6'-​hexahydroxy[1,​1'-​biphenyl]​-​2,​2'-​dicarboxylate] ; D-​Glucose, cyclic 4,​6-​(4,​4',​5,​5',​6,​6'-​hexahydroxy[1,​1'-​biphenyl]​-​2,​2'-​dicarboxylate) (9CI) | 81623-36-1;  81571-73-5;  36378-48-0 | 917.45724; **481.22837** | 300.94851; 419.28163 |
| 37.6 | 1.01 | C_30_H_61_O_17_ (-2.593) | Not match |  | **693.38883**; 761.37703 | 485.31739; 647.00209 |
| 38.16 | 1.92 | C_17_H_12_O_8_ (1.069) | ​Benzopyrano[5,​4,​3-​cde]​[1]​benzopyran-​5,​10-​dione, 2-​hydroxy-​3,​7,​8-​trimethoxy- | 5145-53-9;  1617-49-8 | **343.04701**; 687.10144 | 327.96991; 329.01835 |
| 39.93 | 4.47 | C_42_H_66_O_14_ (-1.803) | β-​D-​Glucopyranosiduronic acid, (3β)​-​17-​carboxy-​28-​norolean-​12-​en-​3-​yl, 6-​β-​D-​glucopyranosyl ester; β-​D-​Glucopyranosiduronic acid, (3β)​-​28-​(β-​D-​glucopyranosyloxy)​-​28-​oxoolean-​12-​en-​3-​yl | 70938-73-7;  51415-02-2 | **793.44205**; 1168.16587 | 585.37848; 747.04155 |
| 40.89 | 6.04 | C_36_H_56_O_12_ (-1.549) | Urs-​12-​ene-​23,​28-​dioic acid, 2,​3,​19-​trihydroxy-​, 28-​β-​D-​glucopyranosyl ester, (2α,​3β,​4α)​- | 95645-51-5 | **679.41061**; 793.44070 | 471.34578; 633.05658 |
| 42.36 | 3.68 | C_35_H_71_O_19_ (0.147) | Not match |  | **795.45965**; 549.34596 | 587.41705; 749.08521 |
| 43.82 | 1.15 | C_35_H_56_O_7_ (1.628) | Urs-​12-​en-​28-​oic acid, 3-​(β-​L-​arabinopyranosyloxy)​-​, (3β)​- | 32180-34-0 | **587.36057**; 293.17638 | 293.03768; 236.03243 |
| 46.15 | 2.25 | C_36_H_58_O_10_ (0.929) | Olean-​12-​en-​28-​oic acid, 2,​3,​19-​trihydroxy-​, β-​D-​glucopyranosyl ester, (2α,​3α,​19α)​- ; Urs-​12-​en-​28-​oic acid, 2,​3,​19-​trihydroxy-​, β-​D-​glucopyranosyl ester, (2α,​3α)​- ; Urs-​12-​en-​28-​oic acid, 2,​3,​19-​trihydroxy-​, β-​D-​glucopyranosyl ester, (2α,​3β)​- ; Urs-​12-​en-​28-​oic acid, 3,​19,​23-​trihydroxy-​, β-​D-​glucopyranosyl ester, (3β,​4α)​- ; | 1118762-14-3;  95298-47-8;  88515-58-6;  42719-32-4 | **649.39665**; 531.33426 | 497.08437; 479.08449 |
| 47.35 | 1.41 | C_31_H_49_O_7_ (1.023) | Not match |  | **533.34940;** 975.69599 | 487.31968; 428.97712 |
| 48.74 | 1.21 | C_30_H_45_O_5_ (1.352) | Not match |  | **485.32860**; 531.33427 | 467.29611; 351.24150 |
| 50.05 | 1.8 | C_37_H_57_O_10_ (2.599) | Not match |  | **661.39832**; 729.38584 | 453.30156; 615.02344 |
| 51.93 | 2.59 | C_20_H_20_O_14_ (2.092) | Urs-​12-​en-​28-​oic acid, 19-​hydroxy-​3,​11-​dioxo- ; D-​Ribofuranose, 2-​C-​[[(3,​4,​5-​trihydroxybenzoyl)​oxy]​methyl]​-​, 1-​(3,​4,​5-​trihydroxybenzoate) ; Benzoic acid, 3,​4-​dihydroxy-​5-​[[6-​O-​(3,​4,​5-​trihydroxybenzoyl)​-​β-​D-​glucopyranosyl]​oxy]​- ; D-​Ribose, 2-​C-​[[(3,​4,​5-​trihydroxybenzoyl)​oxy]​methyl]​-​, 5-​(3,​4,​5-​trihydroxybenzoate) | 142299-63-6;  90275-98-2; 87087-61-4;  469-32-9 | **483.31369**; 529.32048 | 421.30641; 465.22971 |
| 53.74 | 2.54 | C_36_H_55_O_9_ (1.734) | NOT YET ASSIGNED | 1983976-35-7 | **631.38689**; 1171.77440 | 468.95154; 314.97905 |
| 54.39 | 0.68 | C_31_H_47_O_7_ (1.613) | Not match |  | **531.33434**; 485.32872 | 485.31843; 510.97270 |

**Table S3 Positive ESI Mass spectrometry (m/z) analysis of extract CDY3; peaks with >0.5% relative abundance is listed.**

| **RT (min)** | **Relative**  **Abundance** | **Formula (D ppm)** | **Putative Compounds (Scifinder)** | **CAS** | **m/z  ([M+H]- unless indicated)** | **MS^2^** |
| --- | --- | --- | --- | --- | --- | --- |
| 2.91 | 1.92 | C_14_H_23_O_12_ (-2.682) | 1-​Hexacosanol | 506-52-5 | 383.11572; 203.05270 | 202.94936; 220.07121 |
| 3.6 | 0.6 | C_16_H_33_O_18_ (-2.630) | Not match |  | 513.16458; 411.14644 | 415.82797; 318.91721 |
| 10.59 | 0.52 | C_23_H_16_O (-1.202) | 2H-​2,​4a-​(Epoxymethano)​picene (8CI,​9CI) | 169-91-5 | 307.08086; 334.15062 | 138.90411; 150.89515 |
| 11.08 | 1.87 | C_24_H_28_O_2_ (2.933) | Benzoic acid, 4-​[1-​(5,​6,​7,​8-​tetrahydro-​3,​5,​5,​8,​8-​pentamethyl-​2-​naphthalenyl)​ethenyl]​- | 153559-49-0 | 710.21728; 347.09836 | 346.73255; 363.64683 |
| 11.77 | 1.3 | C_28_H_36_O_14_ (-0.110) | D-​Ribofuranoside, methyl 3-​O-​methyl-​2-​C-​[[(3,​4,​5-​trimethoxybenzoyl)​oxy]​methyl]​-​, 5-​(3,​4,​5-​trimethoxybenzoate) | 90276-01-0 | 595.14510; 472.11126 | 426.97310; 246.99897 |
| 12.62 | 0.82 | C_24_H_28_O_21_ (1.599) | Olean-​12-​en-​28-​oic acid, 2,​3,​19-​trihydroxy-​, β-​D-​glucopyranosyl ester, (2α,​3α,​19α)​- | 1118762-14-3 | 652.11335; 483.07732 | 455.17668; 473.05243 |
| 13.82 | 7.12 | C_30_H_27_O_12_ (-1.608) | D-​Ribofuranoside, methyl 3-​O-​methyl-​2-​C-​[[(3,​4,​5-​trimethoxybenzoyl)​oxy]​methyl]​-​, 5-​(3,​4,​5-​trimethoxybenzoate) | 90276-01-0 | 579.14932; 674.10227 | 426.95633; 409.05446 |
| 14.63 | 2.4 | C_15_H_15_O_6_ (-1.969) | Bicyclo[3.3.1]​non-​3-​ene-​2,​9-​dione, 6-​(3,​4-​dihydroxyphenyl)​-​4,​7-​dihydroxy-​, (1R,​5S,​6R,​7S)​- | 52484-79-4 | 291.08629; 652.11388 | 122.89705; 138.88563 |
| 15.42 | 1.38 | C_21_H_23_O_14_ (-2.645) | β-​D-​Glucopyranoside, methyl, 6-​[3,​5-​dihydroxy-​4-​[(3,​4,​5-​trihydroxybenzoyl)​oxy]​benzoate] | 89915-42-4 | 499.10746; 654.13102 | 466.81209; 453.21321 |
| 16.49 | 0.87 | C_41_H_64_O_12_ (0.356) | Ursa-​12,​18-​dien-​28-​oic acid, 3-​(α-​L-​arabinopyranosyloxy)​-​, β-​D-​glucopyranosyl ester, (3β)​- | 435269-07-1 | 747.15288; 467.08120 | 271.00375; 427.07900 |
| 16.96 | 1.22 | C_27_H_24_O_18_ (-3.412) | D-​Ribofuranose, 2-​C-​[[(3,​4,​5-​trihydroxybenzoyl)​oxy]​methyl]​-​, 1,​5-​bis(3,​4,​5-​trihydroxybenzoate) | 90275-99-3 | 637.10200; 291.08587 | 618.95306; 484.97955 |
| 18.09 | 4.13 | C_37_H_31_O_16_ (-1.367) | Benzoic acid, 3,​4,​5-​trihydroxy-​, (2R,​2'R,​3S,​3'S,​4S)​-​2,​2'-​bis(3,​4-​dihydroxyphenyl)​-​3,​3',​4,​4'-​tetrahydro-​3',​5,​5',​7,​7'-​pentahydroxy[4,​8'-​bi-​2H-​1-​benzopyran]​-​3-​yl ester | 86631-41-6 | 731.16021; 441.08183 | 411.04898; 271.00358 |
| 18.77 | 2.71 | C_30_H_42_O_5_ (0.690) | Ursa-​12,​17,​19,​21-​tetraen-​23-​oic acid, 2,​3,​27-​trihydroxy-​, methyl ester, (2α,​3β,​4α)​- | 1172584-63-2 | 484.27695; 467.24981 | 466.41300; 294.85479 |
| 19.93 | 1.17 | C_36_H_58_O_8_ (-1.848) | Urs-​12-​en-​28-​oic acid, 3-​(α-​L-​arabinopyranosyloxy)​-​19-​hydroxy-​, methyl ester, (3β)​- | 35286-61-4 | 619.09238; 806.14185 | 448.97713; 236.93914 |
| 21.6 | 0.91 | C_41_H_70_O_13_ (1.125) | β-​D-​Glucopyranoside, (3β,​6α,​12β)​-​3,​6,​12-​trihydroxydammar-​24-​en-​20-​yl 6-​O-​α-​L-​arabinofuranosyl- | 189513-26-6 | 771.10184; 958.15504 | 304.88551; 260.94737 |
| 22.34 | 0.63 | C_29_H_24_O_12_ (4.288) | 5H-​Benzocyclohepten-​5-​one, 1,​8-​bis[(2R,​3R)​-​3,​4-​dihydro-​3,​5,​7-​trihydroxy-​2H-​1-​benzopyran-​2-​yl]​-​3,​4,​6-​trihydroxy- | 4675-05-7 | 566.42593; 588.41100 | 570.43795; 475.33195 |
| 23.33 | 0.47 | C_35_H_31_O_22_ (-0.930) | β-​D-​Glucopyranoside, methyl, 2,​3,​4,​6-​tetrakis(3,​4,​5-​trihydroxybenzoate) | 52704-75-3 | 803.12995; 820.15855 | 784.91325; 767.86446 |
| 24.24 | 2.19 | C_36_H_56_O_12_ (3.163) | Urs-​12-​ene-​23,​28-​dioic acid, 2,​3,​19-​trihydroxy-​, 28-​β-​D-​glucopyranosyl ester, (2α,​3β,​4α)​- | 95645-51-5 | 679.51117; 340.25952 | 661.48397; 548.42329 |
| 26.5 | 1.89 | C_28_H_46_O (-2.336) | Ergosta-​5,​22-​dien-​3-​ol, (3β,​22E)​- | 474-67-9 | 396.80124; 524.14002 | 345.02811; 506.55090 |
| 28.63 | 3.86 | C_50_H_74_O_15_ (1.059) | β-​D-​Glucopyranoside, (2E)​-​7-​hydroxy-​3,​7-​dimethyl-​2-​octen-​1-​yl 6-​O-​α-​L-​arabinopyranosyl- | 914641-92-2 | 466.26674; 914.50374 | 777.77230; 697.66930 |
| 29.86 | 0.76 | C_22_H_21_O_12_ (1.004) | Benzopyrano[5,​4,​3-​cde]​[1]​benzopyran-​5,​10-​dione, 2,​3,​8-​trimethoxy-​7-​(β-​D-​xylopyranosyloxy)​- | 136133-08-9 | 477.10378; 345.06126 | 345.02944; 373.13441 |
| 30.7 | 1.15 | C_21_H_38_O_11_ (-5.127) | β-​D-​Glucopyranoside, (2E)​-​7-​hydroxy-​3,​7-​dimethyl-​2-​octen-​1-​yl 6-​O-​α-​L-​arabinofuranosyl- | 158306-28-6 | 468.28155; 425.01818 | 345.03591; 383.94340 |
| 32.04 | 2.27 | C_30_H_49_O_5_ (2.739) | 9,​19-​Cyclolanostane-​3,​15,​16-​triol, 16,​23:24,​25-​diepoxy-​, (3β,​15α,​16β,​23R,​24S)​- | 82872-68-2 | 489.35934; 668.43917 | 471.06889; 453.10504 |
| 32.66 | 2.21 | C_30_H_49_O_5_ (-0.612) | 9,​19-​Cyclolanostane-​3,​15,​16-​triol, 16,​23:24,​25-​diepoxy-​, (3β,​15α,​16β,​23R,​24S)​- | 82872-68-3 | 489.35951; 668.43907 | 471.06889; 453.10505 |
| 33.95 | 10.34 | C_35_H_57_O_8_ (-0.369) | Olean-​12-​en-​28-​oic acid, 3-​(α-​L-​arabinopyranosyloxy)​-​19-​hydroxy-​, (3β,​19α)​- | 866216-15-1 | 605.40512; 784.48746 | 455.17252; 473.03498 |
| 37.53 | 0.83 | C_30_H_47_O_5_ (-1.887) | Benzo[3,​4]​-​18-​norandrosta-​3,​5,​15-​triene-​3(2'H)​-​carboxylic acid, 3',​4',​5',​6'-​tetrahydro-​3'-​hydroxy-​15,​17-​bis(hydroxymethyl)​-​3',​4',​9,​14,​17-​pentamethyl-​, (3β,​3'α,​4β,​4'α,​8α,​9β,​10α,​13α,​14β,​17β)​- | 214285-76-4 | 487.34143; 666.42209 | 469.08546; 441.19410 |
| 38.18 | 2.77 | C_17_H_13_O_8_ (2.805) | Benzopyrano[5,​4,​3-​cde]​[1]​benzopyran-​5,​10-​dione, 2-​hydroxy-​3,​7,​8-​trimethoxy- | 1617-49-8 | 345.06201; 706.14248 | 312.99968; 329.99525 |
| 39.93 | 7.48 | C_30_H_62_O (3.943) | 1-​Triacontanol | 593-50-0 | 437.34368; 587.39469 | 391.28337; 191.09438 |
| 40.84 | 6.7 | C_36_H_58_O_10_ (2.314) | Urs-​12-​en-​28-​oic acid, 2,​3,​19-​trihydroxy-​, β-​D-​glucopyranosyl ester, (2α,​3α)​- | 95298-47-8 | 473.36418; 652.44350 | 455.16634; 427.11149 |
| 42.27 | 3.69 | C_30_H_49_O_6_ (0.863) | Urs-​12-​en-​28-​oic acid, 1,​2,​3,​19-​tetrahydroxy-​, (1β,​2α,​3α)​- | 120211-98-5 | 505.35335; 439.35869 | 469.14717; 487.03252 |
| 45.94 | 1.69 | C_35_H_57_O_8_ (-0.667) | Urs-​12-​en-​28-​oic acid, 3-​(α-​L-​arabinopyranosyloxy)​-​19-​hydroxy-​, (3β)​- | 35286-59-0 | 605.40494; 437.34144 | 455.17252; 473.03498 |
| 47.28 | 2.04 | C_30_H_49_O_5_ (-0.122) | Olean-​12-​en-​28-​oic acid, 2,​3,​19-​trihydroxy-​, (2α,​3β,​19α)​- | 31298-06-3 | 489.35794; 471.34803 | 471.06889; 453.10504 |
| 48.7 | 1.43 | C_30_H_48_O_4_ (0.221) | Benzo[3,​4]​-​18-​norandrosta-​3,​5,​15-​triene-​3(2'H)​-​carboxylic acid, 3',​4',​5',​6'-​tetrahydro-​3'-​hydroxy-​15-​(hydroxymethyl)​-​3',​4',​9,​14,​17,​17-​hexamethyl-​, (3β,​3'α,​4β,​4'α,​8α,​9β,​10α,​13α,​14β)​- | 128397-09-1 | 469.33193; 451.32132 | 451.13261; 423.20293 |
| 50.06 | 2.07 | C_36_H_59_O_9_ (1.888) | Olean-​12-​en-​28-​oic acid, 3,​19-​dihydroxy-​, β-​D-​glucopyranosyl ester, (3β,​19α)​- | 155653-86-4 | 634.43193; 455.35338 | 437.12323; 455.03872 |
| 51.93 | 7.1 | C_30_H_45_O_5_ (2.494) | Urs-​12-​en-​28-​oic acid, 19-​hydroxy-​3,​11-​dioxo- | 142299-63-6 | 485.32791; 969.65211 | 466.87360; 296.85126 |
| 52.89 | 1.34 | C_30_H_45_O_5_ (-1.235) | Urs-​12-​en-​28-​oic acid, 19-​hydroxy-​3,​11-​dioxo- | 142299-63-7 | 485.32610; 277.14148 | 466.87360; 296.85127 |
| 53.72 | 0.91 | C_30_H_62_O (0.971) | 1-​Triacontanol | 593-50-0 | 437.34238; 587.39523 | 391.28337; 191.09438 |
| 54.73 | 1.11 | C_30_H_47_O_4_ (0.350) | Benzo[3,​4]​-​18-​norandrosta-​3,​5,​15-​triene-​3(2'H)​-​carboxylic acid, 3',​4',​5',​6'-​tetrahydro-​3'-​hydroxy-​15-​(hydroxymethyl)​-​3',​4',​9,​14,​17,​17-​hexamethyl-​, (3β,​3'α,​4β,​4'α,​8α,​9β,​10α,​13α,​14β)​- | 128397-09-1 | 471.34760; 488.37466 | 453.06941; 425.15645 |
| 56.82 | 0.77 | C_30_H_45_O_4_ (0.714) | Urs-​12-​en-​28-​oic acid, 19-​hydroxy-​3-​oxo- | 13849-90-6 | 469.33212; 491.31797 | 451.13261; 423.20293 |
